# Ethyl 4-hydr­oxy-1-(2-morpholinopro­pano­yl)-2,6-diphenyl-1,2,5,6-tetra­hydro­pyridin-3-carboxyl­ate

**DOI:** 10.1107/S1600536809050259

**Published:** 2009-11-28

**Authors:** G. Aridoss, D. Gayathri, R. Ramachandran, Kwon Taek Lim, Yeon Tae Jeong

**Affiliations:** a Division of Image Science and Information Engineering, Pukyong National University, Busan 608-739, Republic of Korea; b Institute of Structural Biology and Biophysics-2: Molecular Biophysics, Research Centre Jülich, D-52425 Jülich, Germany; c Department of Chemistry, Annamalai University, Annamalai Nagar 608 002, India

## Abstract

In the title compound, C_27_H_32_N_2_O_5_, the morpholine ring adopts a chair conformation with two C atoms deviating by −0.656 (4) and 0.679 (3) Å from the least-squares plane defined by the rest of atoms in the ring. The tetra­hydro­pyridine ring adopts a half-chair conformation. The mol­ecular structure is stabilized by a strong intra­molecular O—H⋯O inter­action, generating an *S*(6) motif. The crystal packing is stabilized by inter­molecular C—H⋯O inter­actions, generating a *C*(7) chain along the *a* axis, and *R*_2_^2^(20) and *R*_4_^4^(20) graph-set motifs.

## Related literature

For related structures, see: Aridoss *et al.* (2009*a* ▶,*b* ▶); Gayathri *et al.* (2008 ▶); Kavitha *et al.* (2007 ▶); Ramachandran *et al.* (2008 ▶); Subha Nandhini *et al.* (2003 ▶). For ring conformational analysis, see: Cremer & Pople (1975 ▶); Nardelli (1983 ▶).
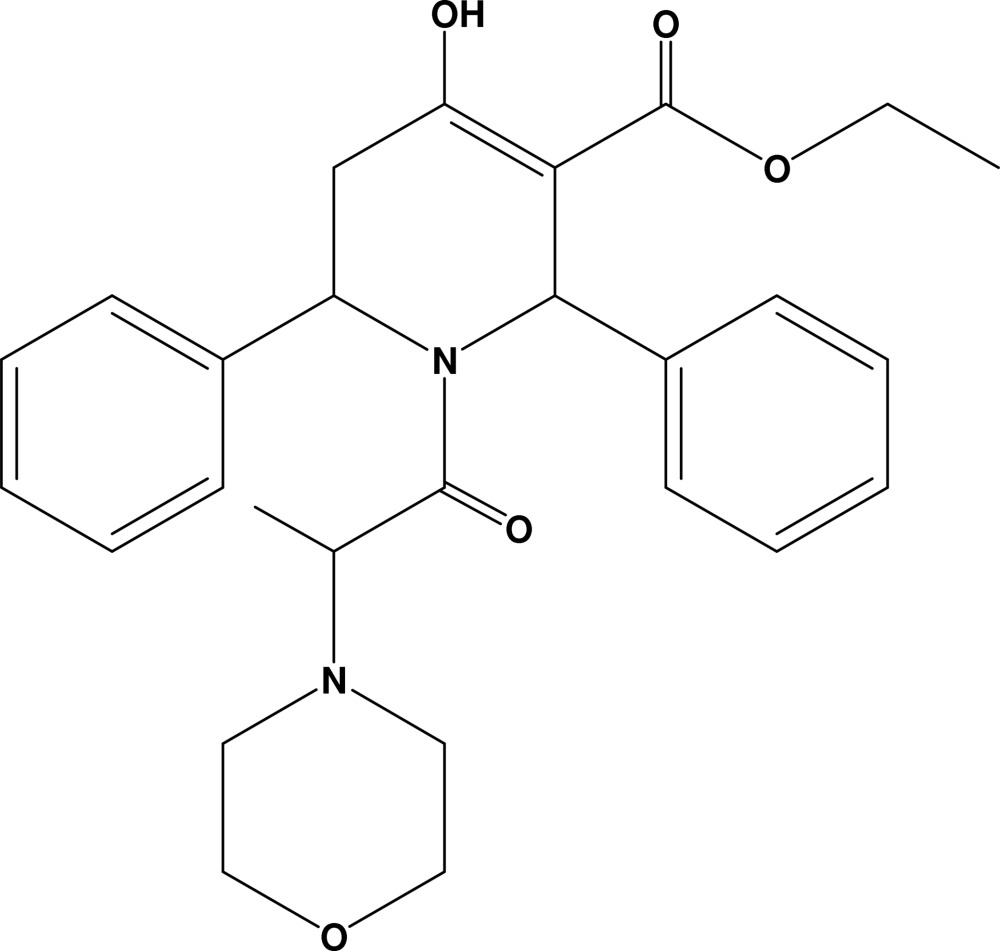


## Experimental

### 

#### Crystal data

C_27_H_32_N_2_O_5_
*M* *_r_* = 464.55
Monoclinic, 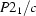

*a* = 8.2251 (2) Å
*b* = 10.6219 (4) Å
*c* = 28.7046 (10) Å
β = 92.375 (2)°
*V* = 2505.66 (14) Å^3^
*Z* = 4
Mo *K*α radiation
μ = 0.09 mm^−1^
*T* = 293 K
0.30 × 0.20 × 0.20 mm

#### Data collection

Bruker Kappa APEXII CCD diffractometer
Absorption correction: multi-scan (**SADABS**; Bruker 1999 ▶) *T*_min_ = 0.975, *T*_max_ = 0.983
22895 measured reflections
4850 independent reflections
3353 reflections with *I* > 2σ(*I*)
*R*_int_ = 0.036

#### Refinement

*R*[*F* ^2^ > 2σ(*F* ^2^)] = 0.052
*wR*(*F* ^2^) = 0.145
*S* = 1.02
4850 reflections
308 parameters
H-atom parameters constrained
Δρ_max_ = 0.29 e Å^−3^
Δρ_min_ = −0.21 e Å^−3^

### 

Data collection: *APEX2* (Bruker, 2004 ▶); cell refinement: *SAINT* (Bruker, 2004 ▶); data reduction: *SAINT*; program(s) used to solve structure: *SIR92* (Altomare *et al.*, 1993 ▶); program(s) used to refine structure: *SHELXL97* (Sheldrick, 2008 ▶); molecular graphics: *PLATON* (Spek, 2009 ▶); software used to prepare material for publication: *SHELXL97*.

## Supplementary Material

Crystal structure: contains datablocks I, global. DOI: 10.1107/S1600536809050259/is2497sup1.cif

Structure factors: contains datablocks I. DOI: 10.1107/S1600536809050259/is2497Isup2.hkl

Additional supplementary materials:  crystallographic information; 3D view; checkCIF report

## Figures and Tables

**Table 1:** Hydrogen-bond geometry (Å, °)

*D*—H⋯*A*	*D*—H	H⋯*A*	*D*⋯*A*	*D*—H⋯*A*
O3—H3*A*⋯O4	0.82	1.83	2.542 (2)	145
C10—H10*A*⋯O1^i^	0.97	2.51	3.311 (3)	140
C11—H11*A*⋯O2^ii^	0.97	2.54	3.222 (3)	127
C26—H26*B*⋯O3^iii^	0.97	2.53	3.470 (4)	163

Symmetry codes: (i) 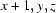; (ii) 
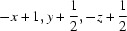
; (iii) 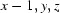.

**Fig. 1.**
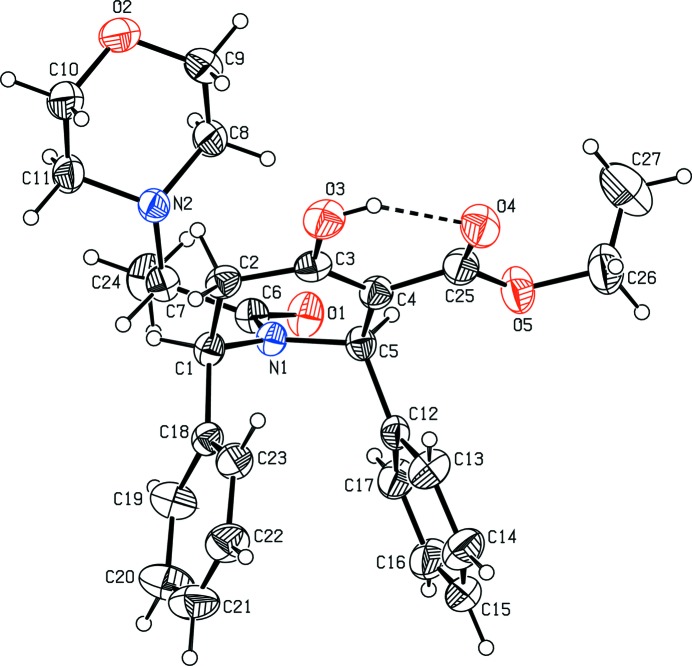
The molecular structure of title compound, showing 30% probability displacement ellipsoids.

**Fig. 2.**
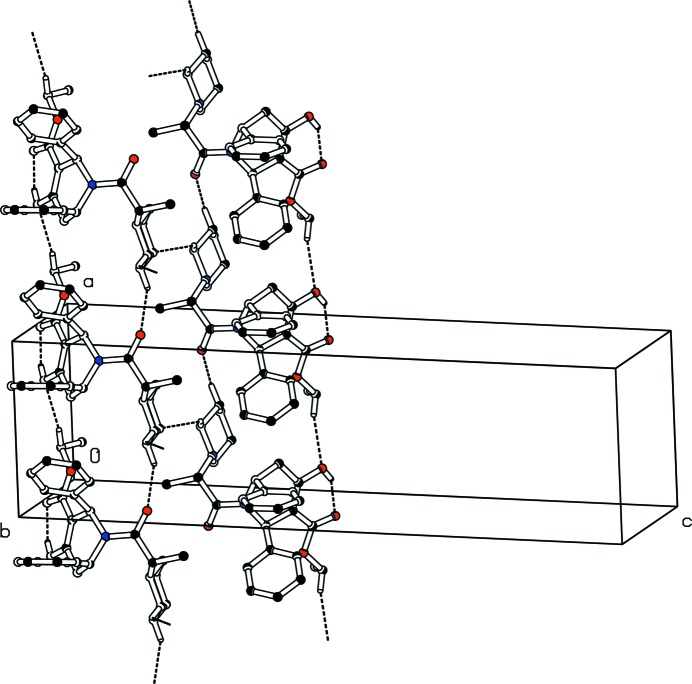
The molecular packing of (I). For clarity, hydrogen atoms which are not involved in hydrogen bonding are omitted.

## supplementary crystallographic information

### Comment

It has been reported earlier that 2,6-diarylpiperidin-4-ones are in rigid chair conformation with equatorial orientation of the two aryl groups (Gayathri *et al.* 2008; Kavitha *et al.* 2007). Upon chloroacetylation (Aridoss *et al.* 2009*b*) or bromoacetylation (Ramachandran *et al.* 2008) of the 2,6-diarylpiperidin-4-one, the normal chair conformation of the piperidone ring was changed into non-chair conformation of its preference. Similarly, bromopropionylation of ethyl 4-oxo-2,6-diphenyl-4-piperidin-3-carboxylate gave ethyl 1-(2-bromopropanoyl)-4-hydroxy-2,6-diphenyl- 1,2,5,6-tetrahydropyridin-3-carboxylate as the product (Aridoss *et al.* 2009*a*) wherein the normal chair conformation of the piperidone ring in starting material (Subha Nandhini *et al.* 2003) was changed into a non-chair conformation. In continuation of this, we report here the crystal structure of the title compound.

The sum of the angles at N1 [359.5 (6)°] and N2 [337.6 (6)°] are in accordance with *sp*^2^ and *sp*^3^ hybridization. The dihedral angle between the two phenyl rings is 35.0 (1)°. The morpholine ring adopts a chair conformation with atoms C9 and C11 deviating by -0.656 (4) and 0.679 (3) Å, respectively, from the least squares plane defined by N2/C8/O2/C10. The tetrahydropyridine ring adopts a half chair conformation. The puckering parameters (Cremer & Pople, 1975) and the smallest displacement asymmetry parameters (Nardelli, 1983) for the morpholine and tetrahydropyridine rings are q_2_ = 0.010 (3), 0.324 (2) Å, q_3_ = -0.572 (3), -0.287 (2) Å; Q_T_ = 0.573 (3), 0.433 (2) Å and θ = 177.8 (3), 131.5 (3)°, respectively.

The molecule is stabilized by a strong O—H···O intramolecular interaction, wherein, atom O3 acts as a donor to O4, generating an S(6) motif. The crystal packing is stabilized by C—H···O intermolecular interactions. Atoms C10 and C26 act as donors to O1 and O3, respectively, each generating a chain of C(7) running along the *a* axis, which in turn generates an *R*_2_^2^(20) graph set motif. Atom C11 acts as a donor to O2 at (1 - *x*, 1/2 + *y*, 1/2 - *z*), generating an *R*_4_^4^(20) graph set motif together with a C10—H10A···O1 intermolecular interaction.

### Experimental

To a solution of morpholine (1 equiv.) and dry K_2_CO_3_ in benzene, ethyl 1-(2-bromopropanoyl)-4-hydroxy-2,6-diphenyl-1,2,5,6-tetrahydropyridin-3- carboxylate (1 equiv.) in benzene (Aridoss *et al.*, 2009*a*) was added slowly over a period of 15 minutes. Later the contents were refluxed over night. After the completion of reaction, the contents were poured into water and extracted twice with ethyl acetate. The combined organic extracts were then washed well with brine and dried over anhydrous sodium sulfate. This upon evaporation, column purification and subsequent recrystallization in distilled ethanol afforded fine white crystals suitable for X-ray diffraction study.

### Refinement

C-bound H atoms were refined using a riding model, with d(C—H) = 0.93 Å and *U*_iso_(H) = 1.2*U*_eq_(C) for aromatic, 0.98 Å and *U*_iso_(H) = 1.2*U*_eq_(C) for CH, 0.97 Å and *U*_iso_(H) = 1.2*U*_eq_(C) for CH_2_, and 0.96 Å and *U*_iso_(H) = 1.5*U*_eq_(C) for CH_3_. The H atom of the OH group was also refined using a riding model, with d(O—H) = 0.82 Å, but the *U*_iso_(H) was refiend freely.

### Crystal data

**Table:**

C_27_H_32_N_2_O_5_	*F*(000) = 992
*M**_r_* = 464.55	*D*_x_ = 1.231 Mg m^−^^3^
Monoclinic, *P*2_1_/*c*	Mo *K*α radiation, λ = 0.71073 Å
Hall symbol: -P 2ybc	Cell parameters from 5113 reflections
*a* = 8.2251 (2) Å	θ = 2.4–23.3°
*b* = 10.6219 (4) Å	µ = 0.09 mm^−^^1^
*c* = 28.7046 (10) Å	*T* = 293 K
β = 92.375 (2)°	Block, colourless
*V* = 2505.66 (14) Å^3^	0.30 × 0.20 × 0.20 mm
*Z* = 4	

### Data collection

**Table:**

Bruker Kappa APEXII CCD diffractometer	4850 independent reflections
Radiation source: fine-focus sealed tube	3353 reflections with *I* > 2σ(*I*)
graphite	*R*_int_ = 0.036
ω and φ scan	θ_max_ = 25.9°, θ_min_ = 2.0°
Absorption correction: multi-scan (*SADABS*; Bruker 1999)	*h* = −10→9
*T*_min_ = 0.975, *T*_max_ = 0.983	*k* = −13→12
22895 measured reflections	*l* = −35→35

### Refinement

**Table:**

Refinement on *F*^2^	Primary atom site location: structure-invariant direct methods
Least-squares matrix: full	Secondary atom site location: difference Fourier map
*R*[*F*^2^ > 2σ(*F*^2^)] = 0.052	Hydrogen site location: inferred from neighbouring sites
*wR*(*F*^2^) = 0.145	H-atom parameters constrained
*S* = 1.02	*w* = 1/[σ^2^(*F*_o_^2^) + (0.0554*P*)^2^ + 1.1339*P*] where *P* = (*F*_o_^2^ + 2*F*_c_^2^)/3
4850 reflections	(Δ/σ)_max_ < 0.001
308 parameters	Δρ_max_ = 0.29 e Å^−^^3^
0 restraints	Δρ_min_ = −0.21 e Å^−^^3^

### Special details

**Table:**

Geometry. All e.s.d.'s (except the e.s.d. in the dihedral angle between two l.s. planes) are estimated using the full covariance matrix. The cell e.s.d.'s are taken into account individually in the estimation of e.s.d.'s in distances, angles and torsion angles; correlations between e.s.d.'s in cell parameters are only used when they are defined by crystal symmetry. An approximate (isotropic) treatment of cell e.s.d.'s is used for estimating e.s.d.'s involving l.s. planes.
Refinement. Refinement of *F*^2^ against ALL reflections. The weighted *R*-factor *wR* and goodness of fit *S* are based on *F*^2^, conventional *R*-factors *R* are based on *F*, with *F* set to zero for negative *F*^2^. The threshold expression of *F*^2^ > σ(*F*^2^) is used only for calculating *R*-factors(gt) *etc*. and is not relevant to the choice of reflections for refinement. *R*-factors based on *F*^2^ are statistically about twice as large as those based on *F*, and *R*- factors based on ALL data will be even larger.

### Fractional atomic coordinates and isotropic or equivalent isotropic displacement parameters (Å^2^)

**Table:**

	*x*	*y*	*z*	*U*_iso_*/*U*_eq_	
O1	−0.09176 (19)	0.60293 (18)	0.27695 (5)	0.0644 (5)	
N2	0.2969 (2)	0.54061 (17)	0.28207 (6)	0.0498 (5)	
C1	0.2028 (2)	0.6983 (2)	0.36715 (7)	0.0444 (5)	
H1A	0.2803	0.7179	0.3432	0.053*	
C2	0.2787 (3)	0.5948 (2)	0.39704 (8)	0.0509 (5)	
H2A	0.3412	0.5401	0.3775	0.061*	
H2B	0.3535	0.6323	0.4201	0.061*	
O2	0.4987 (2)	0.32639 (18)	0.27766 (9)	0.0972 (7)	
O3	0.2277 (2)	0.44389 (18)	0.45418 (6)	0.0688 (5)	
H3A	0.1594	0.3950	0.4637	0.118 (15)*	
C3	0.1586 (3)	0.5184 (2)	0.42121 (7)	0.0502 (5)	
O4	−0.0581 (2)	0.36103 (18)	0.46520 (6)	0.0758 (5)	
C4	−0.0018 (3)	0.5187 (2)	0.40991 (7)	0.0491 (5)	
O5	−0.2586 (2)	0.43166 (18)	0.41704 (7)	0.0763 (5)	
C5	−0.0748 (2)	0.6014 (2)	0.37157 (7)	0.0445 (5)	
H5A	−0.1409	0.5455	0.3513	0.053*	
N1	0.05448 (19)	0.65010 (16)	0.34259 (5)	0.0433 (4)	
C6	0.0380 (3)	0.6339 (2)	0.29554 (7)	0.0486 (5)	
C7	0.1903 (3)	0.6443 (2)	0.26673 (7)	0.0509 (5)	
H7A	0.2453	0.7233	0.2753	0.061*	
C8	0.2302 (3)	0.4162 (2)	0.27172 (10)	0.0669 (7)	
H8A	0.2218	0.4036	0.2382	0.080*	
H8B	0.1219	0.4096	0.2837	0.080*	
C9	0.3375 (3)	0.3183 (3)	0.29354 (13)	0.0853 (9)	
H9A	0.3400	0.3282	0.3272	0.102*	
H9B	0.2933	0.2356	0.2861	0.102*	
C10	0.5629 (3)	0.4474 (3)	0.28766 (12)	0.0811 (9)	
H10A	0.6724	0.4527	0.2765	0.097*	
H10B	0.5690	0.4600	0.3212	0.097*	
C11	0.4610 (3)	0.5486 (2)	0.26541 (9)	0.0634 (7)	
H11A	0.5074	0.6303	0.2731	0.076*	
H11B	0.4584	0.5390	0.2318	0.076*	
C12	−0.1901 (2)	0.7031 (2)	0.38781 (7)	0.0454 (5)	
C13	−0.2138 (3)	0.7287 (3)	0.43393 (8)	0.0654 (7)	
H13A	−0.1534	0.6855	0.4569	0.078*	
C14	−0.3262 (3)	0.8179 (3)	0.44672 (11)	0.0777 (8)	
H14A	−0.3411	0.8335	0.4781	0.093*	
C15	−0.4147 (3)	0.8827 (3)	0.41383 (12)	0.0761 (8)	
H15A	−0.4900	0.9427	0.4225	0.091*	
C16	−0.3922 (3)	0.8588 (3)	0.36786 (12)	0.0791 (8)	
H16A	−0.4518	0.9034	0.3451	0.095*	
C17	−0.2821 (3)	0.7691 (3)	0.35491 (9)	0.0647 (7)	
H17A	−0.2696	0.7528	0.3234	0.078*	
C18	0.1703 (2)	0.8208 (2)	0.39239 (7)	0.0456 (5)	
C19	0.1431 (3)	0.9284 (2)	0.36661 (9)	0.0683 (7)	
H19A	0.1400	0.9239	0.3342	0.082*	
C20	0.1205 (4)	1.0428 (3)	0.38800 (12)	0.0894 (10)	
H20A	0.1021	1.1148	0.3701	0.107*	
C21	0.1254 (4)	1.0502 (3)	0.43555 (12)	0.0841 (9)	
H21A	0.1099	1.1274	0.4500	0.101*	
C22	0.1524 (3)	0.9460 (3)	0.46159 (9)	0.0727 (8)	
H22A	0.1563	0.9515	0.4940	0.087*	
C23	0.1744 (3)	0.8312 (2)	0.44022 (8)	0.0577 (6)	
H23A	0.1923	0.7598	0.4584	0.069*	
C24	0.1449 (3)	0.6501 (3)	0.21484 (8)	0.0750 (8)	
H24A	0.2419	0.6567	0.1976	0.113*	
H24B	0.0771	0.7222	0.2086	0.113*	
H24C	0.0870	0.5750	0.2057	0.113*	
C25	−0.1051 (3)	0.4311 (2)	0.43333 (9)	0.0593 (6)	
C26	−0.3741 (4)	0.3489 (3)	0.43944 (13)	0.0938 (10)	
H26A	−0.3403	0.3369	0.4719	0.113*	
H26B	−0.4810	0.3875	0.4383	0.113*	
C27	−0.3819 (6)	0.2296 (4)	0.41630 (19)	0.1500 (18)	
H27A	−0.4578	0.1761	0.4313	0.225*	
H27B	−0.2762	0.1912	0.4177	0.225*	
H27C	−0.4169	0.2416	0.3843	0.225*	

### Atomic displacement parameters (Å^2^)

**Table:**

	*U*^11^	*U*^22^	*U*^33^	*U*^12^	*U*^13^	*U*^23^
O1	0.0516 (9)	0.0898 (13)	0.0509 (9)	−0.0112 (9)	−0.0078 (7)	−0.0060 (8)
N2	0.0478 (10)	0.0492 (11)	0.0528 (10)	−0.0081 (8)	0.0058 (8)	−0.0110 (8)
C1	0.0400 (11)	0.0524 (13)	0.0408 (10)	−0.0027 (9)	0.0002 (8)	−0.0027 (9)
C2	0.0457 (12)	0.0547 (13)	0.0518 (12)	0.0047 (10)	−0.0029 (9)	−0.0057 (10)
O2	0.0619 (12)	0.0616 (12)	0.169 (2)	0.0010 (9)	0.0195 (12)	−0.0312 (13)
O3	0.0628 (10)	0.0734 (12)	0.0696 (11)	0.0181 (10)	−0.0053 (9)	0.0206 (9)
C3	0.0548 (13)	0.0490 (13)	0.0466 (12)	0.0115 (10)	−0.0001 (10)	−0.0004 (10)
O4	0.0742 (12)	0.0752 (12)	0.0780 (12)	0.0057 (10)	0.0046 (9)	0.0336 (10)
C4	0.0472 (12)	0.0507 (13)	0.0493 (12)	0.0049 (10)	0.0022 (9)	0.0051 (10)
O5	0.0573 (10)	0.0811 (13)	0.0902 (13)	−0.0100 (9)	0.0009 (9)	0.0335 (10)
C5	0.0411 (11)	0.0482 (12)	0.0438 (11)	−0.0037 (9)	−0.0013 (9)	0.0040 (9)
N1	0.0397 (9)	0.0502 (10)	0.0399 (9)	−0.0025 (8)	−0.0006 (7)	−0.0016 (8)
C6	0.0494 (12)	0.0520 (13)	0.0439 (11)	−0.0026 (10)	−0.0020 (9)	−0.0026 (10)
C7	0.0557 (13)	0.0551 (14)	0.0420 (11)	−0.0060 (11)	0.0029 (9)	−0.0028 (10)
C8	0.0549 (14)	0.0582 (15)	0.0883 (18)	−0.0127 (12)	0.0102 (13)	−0.0190 (14)
C9	0.0709 (18)	0.0493 (16)	0.137 (3)	−0.0089 (13)	0.0176 (18)	−0.0140 (17)
C10	0.0524 (15)	0.0664 (18)	0.125 (3)	−0.0077 (13)	0.0122 (15)	−0.0193 (17)
C11	0.0523 (13)	0.0639 (16)	0.0748 (16)	−0.0151 (12)	0.0146 (12)	−0.0146 (13)
C12	0.0364 (10)	0.0493 (12)	0.0505 (12)	−0.0039 (9)	0.0012 (9)	0.0070 (10)
C13	0.0607 (14)	0.0823 (18)	0.0530 (13)	0.0145 (13)	−0.0005 (11)	−0.0028 (13)
C14	0.0641 (16)	0.093 (2)	0.0767 (18)	0.0074 (16)	0.0106 (14)	−0.0208 (16)
C15	0.0513 (15)	0.0607 (17)	0.117 (3)	0.0051 (12)	0.0178 (15)	−0.0037 (17)
C16	0.0612 (16)	0.078 (2)	0.099 (2)	0.0209 (14)	0.0122 (15)	0.0306 (17)
C17	0.0573 (14)	0.0737 (17)	0.0636 (15)	0.0108 (13)	0.0070 (11)	0.0185 (13)
C18	0.0378 (11)	0.0509 (13)	0.0476 (12)	−0.0014 (9)	−0.0027 (9)	−0.0040 (10)
C19	0.0879 (19)	0.0577 (16)	0.0577 (14)	0.0038 (13)	−0.0153 (13)	−0.0019 (12)
C20	0.118 (3)	0.0547 (17)	0.093 (2)	0.0152 (16)	−0.0289 (19)	−0.0008 (16)
C21	0.091 (2)	0.0642 (19)	0.095 (2)	0.0138 (16)	−0.0131 (17)	−0.0268 (17)
C22	0.0827 (19)	0.075 (2)	0.0602 (15)	0.0021 (15)	0.0043 (13)	−0.0204 (15)
C23	0.0636 (14)	0.0596 (15)	0.0502 (13)	−0.0008 (12)	0.0064 (11)	−0.0055 (11)
C24	0.0797 (18)	0.101 (2)	0.0441 (13)	−0.0055 (16)	0.0046 (12)	0.0004 (14)
C25	0.0559 (14)	0.0597 (15)	0.0625 (14)	0.0066 (12)	0.0047 (11)	0.0135 (12)
C26	0.0666 (18)	0.096 (3)	0.119 (3)	−0.0084 (17)	0.0110 (17)	0.041 (2)
C27	0.161 (4)	0.083 (3)	0.206 (5)	−0.035 (3)	0.016 (4)	0.014 (3)

### Geometric parameters (Å, °)

**Table:**

O1—C6	1.219 (2)	C10—H10B	0.9700
N2—C11	1.453 (3)	C11—H11A	0.9700
N2—C8	1.457 (3)	C11—H11B	0.9700
N2—C7	1.464 (3)	C12—C13	1.373 (3)
C1—N1	1.475 (2)	C12—C17	1.377 (3)
C1—C2	1.513 (3)	C13—C14	1.384 (4)
C1—C18	1.518 (3)	C13—H13A	0.9300
C1—H1A	0.9800	C14—C15	1.356 (4)
C2—C3	1.474 (3)	C14—H14A	0.9300
C2—H2A	0.9700	C15—C16	1.364 (4)
C2—H2B	0.9700	C15—H15A	0.9300
O2—C10	1.414 (3)	C16—C17	1.376 (4)
O2—C9	1.423 (3)	C16—H16A	0.9300
O3—C3	1.342 (3)	C17—H17A	0.9300
O3—H3A	0.8200	C18—C19	1.375 (3)
C3—C4	1.345 (3)	C18—C23	1.376 (3)
O4—C25	1.229 (3)	C19—C20	1.378 (4)
C4—C25	1.445 (3)	C19—H19A	0.9300
C4—C5	1.513 (3)	C20—C21	1.366 (4)
O5—C25	1.328 (3)	C20—H20A	0.9300
O5—C26	1.463 (3)	C21—C22	1.349 (4)
C5—N1	1.471 (2)	C21—H21A	0.9300
C5—C12	1.524 (3)	C22—C23	1.380 (3)
C5—H5A	0.9800	C22—H22A	0.9300
N1—C6	1.363 (3)	C23—H23A	0.9300
C6—C7	1.533 (3)	C24—H24A	0.9600
C7—C24	1.522 (3)	C24—H24B	0.9600
C7—H7A	0.9800	C24—H24C	0.9600
C8—C9	1.486 (4)	C26—C27	1.430 (5)
C8—H8A	0.9700	C26—H26A	0.9700
C8—H8B	0.9700	C26—H26B	0.9700
C9—H9A	0.9700	C27—H27A	0.9600
C9—H9B	0.9700	C27—H27B	0.9600
C10—C11	1.491 (4)	C27—H27C	0.9600
C10—H10A	0.9700		
			
C11—N2—C8	109.46 (18)	C10—C11—H11A	109.8
C11—N2—C7	114.22 (18)	N2—C11—H11B	109.8
C8—N2—C7	113.89 (18)	C10—C11—H11B	109.8
N1—C1—C2	109.42 (17)	H11A—C11—H11B	108.3
N1—C1—C18	111.54 (16)	C13—C12—C17	117.7 (2)
C2—C1—C18	115.37 (17)	C13—C12—C5	123.4 (2)
N1—C1—H1A	106.7	C17—C12—C5	118.8 (2)
C2—C1—H1A	106.7	C12—C13—C14	120.9 (2)
C18—C1—H1A	106.7	C12—C13—H13A	119.5
C3—C2—C1	113.42 (17)	C14—C13—H13A	119.5
C3—C2—H2A	108.9	C15—C14—C13	120.6 (3)
C1—C2—H2A	108.9	C15—C14—H14A	119.7
C3—C2—H2B	108.9	C13—C14—H14A	119.7
C1—C2—H2B	108.9	C14—C15—C16	119.3 (3)
H2A—C2—H2B	107.7	C14—C15—H15A	120.4
C10—O2—C9	109.6 (2)	C16—C15—H15A	120.4
C3—O3—H3A	109.5	C15—C16—C17	120.5 (3)
O3—C3—C4	123.6 (2)	C15—C16—H16A	119.8
O3—C3—C2	112.59 (19)	C17—C16—H16A	119.8
C4—C3—C2	123.7 (2)	C12—C17—C16	121.1 (2)
C3—C4—C25	118.4 (2)	C12—C17—H17A	119.5
C3—C4—C5	122.17 (19)	C16—C17—H17A	119.5
C25—C4—C5	119.27 (19)	C19—C18—C23	117.8 (2)
C25—O5—C26	117.9 (2)	C19—C18—C1	118.91 (19)
N1—C5—C4	109.94 (16)	C23—C18—C1	123.2 (2)
N1—C5—C12	113.43 (17)	C18—C19—C20	121.0 (2)
C4—C5—C12	114.99 (17)	C18—C19—H19A	119.5
N1—C5—H5A	105.9	C20—C19—H19A	119.5
C4—C5—H5A	105.9	C21—C20—C19	119.8 (3)
C12—C5—H5A	105.9	C21—C20—H20A	120.1
C6—N1—C5	118.14 (16)	C19—C20—H20A	120.1
C6—N1—C1	124.31 (17)	C22—C21—C20	120.2 (3)
C5—N1—C1	117.05 (15)	C22—C21—H21A	119.9
O1—C6—N1	121.15 (19)	C20—C21—H21A	119.9
O1—C6—C7	120.23 (19)	C21—C22—C23	120.0 (3)
N1—C6—C7	118.41 (18)	C21—C22—H22A	120.0
N2—C7—C24	116.4 (2)	C23—C22—H22A	120.0
N2—C7—C6	106.06 (17)	C18—C23—C22	121.1 (2)
C24—C7—C6	110.97 (18)	C18—C23—H23A	119.5
N2—C7—H7A	107.7	C22—C23—H23A	119.5
C24—C7—H7A	107.7	C7—C24—H24A	109.5
C6—C7—H7A	107.7	C7—C24—H24B	109.5
N2—C8—C9	109.7 (2)	H24A—C24—H24B	109.5
N2—C8—H8A	109.7	C7—C24—H24C	109.5
C9—C8—H8A	109.7	H24A—C24—H24C	109.5
N2—C8—H8B	109.7	H24B—C24—H24C	109.5
C9—C8—H8B	109.7	O4—C25—O5	122.0 (2)
H8A—C8—H8B	108.2	O4—C25—C4	124.3 (2)
O2—C9—C8	111.6 (2)	O5—C25—C4	113.7 (2)
O2—C9—H9A	109.3	C27—C26—O5	110.2 (3)
C8—C9—H9A	109.3	C27—C26—H26A	109.6
O2—C9—H9B	109.3	O5—C26—H26A	109.6
C8—C9—H9B	109.3	C27—C26—H26B	109.6
H9A—C9—H9B	108.0	O5—C26—H26B	109.6
O2—C10—C11	111.7 (2)	H26A—C26—H26B	108.1
O2—C10—H10A	109.3	C26—C27—H27A	109.5
C11—C10—H10A	109.3	C26—C27—H27B	109.5
O2—C10—H10B	109.3	H27A—C27—H27B	109.5
C11—C10—H10B	109.3	C26—C27—H27C	109.5
H10A—C10—H10B	107.9	H27A—C27—H27C	109.5
N2—C11—C10	109.3 (2)	H27B—C27—H27C	109.5
N2—C11—H11A	109.8		
			
N1—C1—C2—C3	−40.7 (2)	C9—O2—C10—C11	−58.4 (3)
C18—C1—C2—C3	86.0 (2)	C8—N2—C11—C10	−57.6 (3)
C1—C2—C3—O3	−167.93 (18)	C7—N2—C11—C10	173.3 (2)
C1—C2—C3—C4	15.2 (3)	O2—C10—C11—N2	58.9 (3)
O3—C3—C4—C25	−2.2 (3)	N1—C5—C12—C13	−120.7 (2)
C2—C3—C4—C25	174.4 (2)	C4—C5—C12—C13	7.0 (3)
O3—C3—C4—C5	−178.2 (2)	N1—C5—C12—C17	62.6 (3)
C2—C3—C4—C5	−1.6 (3)	C4—C5—C12—C17	−169.6 (2)
C3—C4—C5—N1	14.9 (3)	C17—C12—C13—C14	0.0 (4)
C25—C4—C5—N1	−161.11 (19)	C5—C12—C13—C14	−176.7 (2)
C3—C4—C5—C12	−114.6 (2)	C12—C13—C14—C15	−0.5 (4)
C25—C4—C5—C12	69.4 (3)	C13—C14—C15—C16	0.2 (4)
C4—C5—N1—C6	127.7 (2)	C14—C15—C16—C17	0.6 (4)
C12—C5—N1—C6	−102.0 (2)	C13—C12—C17—C16	0.9 (4)
C4—C5—N1—C1	−44.5 (2)	C5—C12—C17—C16	177.7 (2)
C12—C5—N1—C1	85.8 (2)	C15—C16—C17—C12	−1.2 (4)
C2—C1—N1—C6	−113.0 (2)	N1—C1—C18—C19	−71.5 (2)
C18—C1—N1—C6	118.1 (2)	C2—C1—C18—C19	162.9 (2)
C2—C1—N1—C5	58.7 (2)	N1—C1—C18—C23	111.8 (2)
C18—C1—N1—C5	−70.1 (2)	C2—C1—C18—C23	−13.8 (3)
C5—N1—C6—O1	14.1 (3)	C23—C18—C19—C20	−0.1 (4)
C1—N1—C6—O1	−174.3 (2)	C1—C18—C19—C20	−177.0 (3)
C5—N1—C6—C7	−160.63 (18)	C18—C19—C20—C21	0.2 (5)
C1—N1—C6—C7	11.0 (3)	C19—C20—C21—C22	0.1 (5)
C11—N2—C7—C24	67.5 (3)	C20—C21—C22—C23	−0.4 (5)
C8—N2—C7—C24	−59.3 (3)	C19—C18—C23—C22	−0.1 (3)
C11—N2—C7—C6	−168.57 (18)	C1—C18—C23—C22	176.6 (2)
C8—N2—C7—C6	64.6 (2)	C21—C22—C23—C18	0.4 (4)
O1—C6—C7—N2	−110.6 (2)	C26—O5—C25—O4	2.3 (4)
N1—C6—C7—N2	64.2 (2)	C26—O5—C25—C4	−178.2 (2)
O1—C6—C7—C24	16.7 (3)	C3—C4—C25—O4	4.8 (4)
N1—C6—C7—C24	−168.6 (2)	C5—C4—C25—O4	−179.1 (2)
C11—N2—C8—C9	57.5 (3)	C3—C4—C25—O5	−174.8 (2)
C7—N2—C8—C9	−173.2 (2)	C5—C4—C25—O5	1.4 (3)
C10—O2—C9—C8	58.0 (3)	C25—O5—C26—C27	−91.5 (4)
N2—C8—C9—O2	−58.1 (3)		

### Hydrogen-bond geometry (Å, °)

**Table:**

*D*—H···*A*	*D*—H	H···*A*	*D*···*A*	*D*—H···*A*
O3—H3A···O4	0.82	1.83	2.542 (2)	145
C10—H10A···O1^i^	0.97	2.51	3.311 (3)	140
C11—H11A···O2^ii^	0.97	2.54	3.222 (3)	127
C26—H26B···O3^iii^	0.97	2.53	3.470 (4)	163

Symmetry codes: (i) *x*+1, *y*, *z*; (ii) −*x*+1, *y*+1/2, −*z*+1/2; (iii) *x*−1, *y*, *z*.
